# Development of Ag–In Alloy Pastes by Mechanical Alloying for Die Attachment of High-Power Semiconductor Devices

**DOI:** 10.3390/ma15041397

**Published:** 2022-02-14

**Authors:** Chin-Hao Tsai, Wei-Chen Huang, Chengheng Robert Kao

**Affiliations:** 1 Department of Materials Science and Engineering, National Taiwan University, Taipei 10617, Taiwan; f07527060@ntu.edu.tw (C.-H.T.); f08527014@ntu.edu.tw (W.-C.H.); 2 Advanced Research Center for Green Materials Science & Technology, National Taiwan University, Taipei 10617, Taiwan

**Keywords:** Ag–In alloy pastes, mechanical alloying, power semiconductor packaging, die attachment, mechanical properties, oxidation mechanism

## Abstract

Sintered silver paste is widely used as the die-attachment material for power semiconductors. However, sintered silver joints encounter problems, such as severe coarsening of sintered pores and oxidation issues, in harsh high-temperature environments. These lead to the deterioration of the die-attachment joints. In this paper, a novel method of sintering silver joints is demonstrated, where silver–indium alloy paste is used to improve the reliability of sintered Ag joints. The silver–indium (Ag–In) alloy paste was fabricated through mechanical alloying using the ball-milling technique. The well-bonded sintered Ag–In alloy joints inhibited pore coarsening better than pure sintered Ag joints and significantly enhanced the mechanical properties at high operating temperatures. Lastly, an oxidation mechanism for the sintered joint was proposed, and strategies to prevent such high-temperature oxidation were discussed.

## 1. Introduction

In the age of pursuing energy savings and reducing carbon emissions, the popularization of electric vehicles (EVs) has become the primary objective of next-generation transportation technology because the use of EVs can significantly reduce carbon emissions, which will diminish the greenhouse effect and global warming. Consequently, many countries advocate that internal combustion engine vehicles should be phased out and replaced by EVs or hybrid EVs within the next decade [1]. Therefore, it is important to accelerate the development of EVs and enhance the energy conversion efficiency of inverters, the power module responsible for switching between alternating current and direct current power in EVs.

Inverters need to withstand a considerable amount of current in EVs, which leads to a substantial amount of heat generation and a harsh operating temperature of above 200 °C [2,3]. Given that Si-based semiconductors become conductive and can fail at such high temperatures, wide-bandgap (WBG) semiconductors are better suited for use in inverter power chips because of their superior properties compared to Si-based semiconductors [4,5,6]. Silicon carbide (SiC) and gallium nitride (GaN) are two promising WBG semiconductors for application in EV power modules because they exhibit high breakdown voltage, high switching frequency, low switching losses, and low power conversion losses while operating above 200 °C [7,8,9]. Therefore, WBG semiconductor-based power chips increase the horsepower of EVs and simultaneously extend the operating duration owing to their low power consumption.

For power chips to function well, packaging materials with high-temperature reliability are essential for the die attachment, and they should provide power chips with excellent mechanical support and heat dissipation. However, the conventional Pb-free Sn-based packaging materials used in consumer electronics cannot withstand the high temperatures generated during power semiconductor operation due to their intrinsically low melting points and creep resistance [10,11,12]. Therefore, developing reliable die-attachment materials for high-temperature applications is crucial for EV power modules. Ag paste sintering is a die-attachment packaging technology that has been widely used in power modules because sintered silver joints possess outstanding mechanical reliability and heat dissipation properties at high operating temperatures. Nevertheless, sintered Ag joints have challenging Cu substrate oxidation problems that need to be resolved. It was demonstrated that the Cu oxide layers formed at operating temperatures above 200 °C deteriorated the mechanical strength of sintered Ag joints [13,14,15,16,17]. Furthermore, coarsening of sintered pores in high-temperature environments also resulted in reliability issues. To address the aforementioned issues, Lee and coworkers pointed out that a joint comprising Ag–In intermetallic compounds (IMCs) exhibited excellent properties compared to pure Ag joints [18,19]. Moreover, our previous studies have proven that the addition of In to sintered Ag joints has positive effects on the sintered joint [20,21,22,23,24,25]. The sintered Ag–In joint produced via the transient liquid phase (TLP) bonding reaction between the Ag paste and In foil significantly improved the mechanical properties and reduced oxidation problems of the sintered joints at high temperatures. However, the In foils used for TLP bonding are too soft and fragile, leading to handling difficulties during the fabrication process. The additional step of adding In foil also increases the production costs. Hence, the fabrication process for the sintered Ag–In joint using In foil is not fully compatible with large-scale manufacturing. Furthermore, the sintered Ag–In joint produced via the TLP reaction needed to undergo a long-term phase transformation from brittle Ag–In IMCs to a ductile Ag–In solid solution to achieve the optimal mechanical properties and compositional homogenization [24,25]. Consequently, despite the excellent properties of sintered Ag–In joints, it is not practical to use In foil in industrial applications because it cannot be mass-produced, which restricts the application of sintered Ag–In joints.

Herein, a novel method of directly sintering the Ag–In alloy pastes using a homogeneous solid solution is proposed. Through the employment of the Ag–In alloy paste, the additional step of adding In foil can be eliminated, thereby simplifying the fabrication process and bringing sintered Ag–In joints closer to practical industrial application. Such Ag–In alloy pastes can retain the benefits of In foils but at much-reduced manufacturing cost. A proper powder production method needs to be developed for the fabrication of homogeneous Ag–In alloy pastes. Powder production methods such as gas and water atomization are common in industrial applications because they can mass-produce metal and alloy powders [26,27]. However, gas and water atomization are not suitable for the early stages of research and development in Ag–In alloy powders because of the large quantities of raw material required. Therefore, the focus should be on testing the feasibility and optimizing the composition of the Ag–In alloy paste. Hence, this study uses mechanical alloying by the ball-milling technique to produce small quantities of Ag–In alloy powders [28]. In this way, various Ag–In alloy powders can be made and compared to determine the optimal composition.

In this study, a novel Ag–In alloy paste was developed by directly alloying In with Ag powder through the ball-milling process and mixing with an organic solvent. The Ag–In alloy paste was utilized to fabricate sintered joints by a hot-pressing process. In addition, we compared the bonding and sintering results of the fabricated Ag–In alloy joint to the pure Ag joint. Furthermore, the high-temperature reliability of both sintered joints was evaluated using high-temperature storage (HTS) and die shear tests, after which the microstructure and phase were investigated. Moreover, the correlation between the mechanical properties of both sintered joints during HTS was established. Lastly, the oxidation mechanism in the sintered joint and strategies to prevent oxidation were discussed.

## 2. Experimental

### 2.1. Ball-Milling Process for the Fabrication of Ag and Ag–In Alloy Pastes

**Figure 1:**
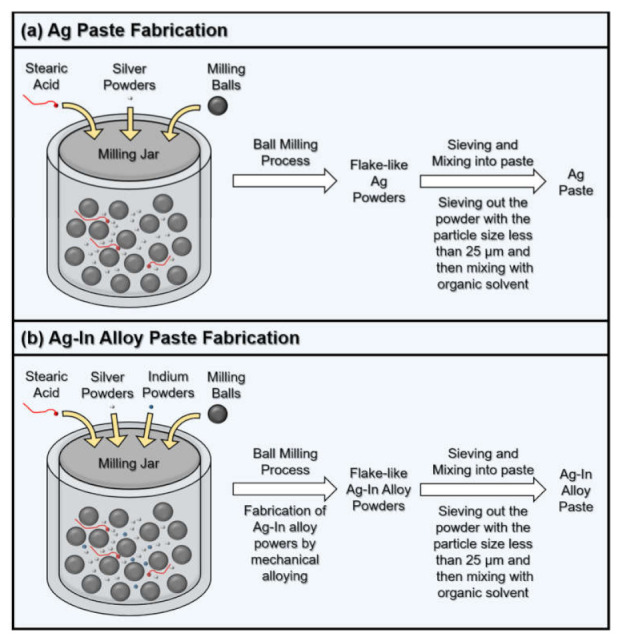
Schematic of the (**a**) Ag and (**b**) Ag–In alloy paste fabrication processes.

Figure 1 shows a schematic of the Ag and Ag–In alloy paste fabrication processes. A high-energy planetary ball-milling machine (Pulverisette 7 Premium Line, Fritsch, Idar-Oberstein, Germany) with two atmosphere-controlled milling jars (Fritsch, Idar-Oberstein, Germany) was employed for the production of the Ag and Ag–In alloy powders. Initially, 10 g of Ag powder with an average particle size of 150 µm, 75 g of zirconium dioxide (ZrO_2_) grinding balls, and 0.2 g of stearic acid were mixed into one of the ball-milling jars, as illustrated in Figure 1a. In the other ball-milling jar, 8 g of Ag powder and 2 g of In powder with an average particle size of 150 µm, 75 g of ZrO_2_ grinding balls, and 0.2 g of stearic acid were mixed, as illustrated in Figure 1b. ZrO_2_ with a diameter of 5 mm was chosen for the grinding balls because ZrO_2_ ceramic is less prone to metal powder contamination during ball-milling. Stearic acid was added to inhibit the cold welding and agglomeration of ductile Ag and In powders during the high-energy ball-milling process. Furthermore, the atmosphere in both ball-milling jars was replaced with high-purity argon gas to prevent oxidation of the Ag and In powders. The milling speed in both jars was 600 rpm for 10 h, after which the jars were opened in a nitrogen glove box (Younme Technology Company, Taoyuan, Taiwan) with an oxygen concentration of less than 0.1 ppm. Then, the ball-milled Ag and Ag–In alloy powders were passed through a 500-mesh sieve. After sieving, Ag and Ag–In alloy powders with particle sizes less than 25 µm were homogeneously mixed with a polymer solvent to complete the preparation of the pastes.

### 2.2. Experimental Bonding Procedure

**Figure 2:**
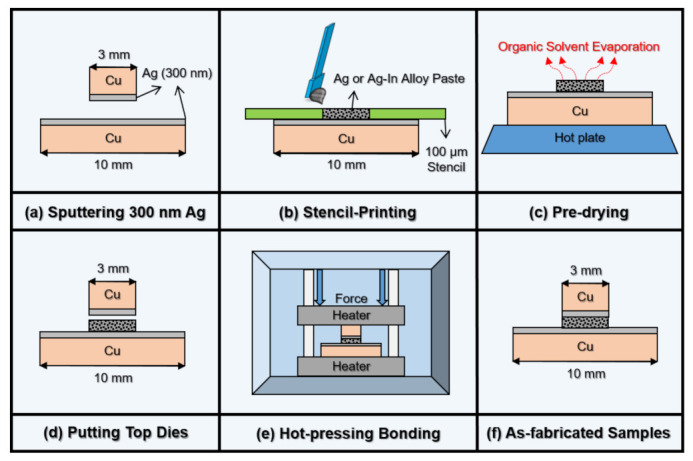
Flowchart of the sintered Ag and Ag–In alloy joint bonding processes. (**a**) Sputtering 300 nm Ag layer on Cu substrates as surface finish metallization, (**b**) printing 100 μm Ag or Ag-In alloy pastes in thickness on lower substrates, (**c**) pre-drying the pastes on a hot plate, (**d**) placing top dies on the pastes, (**e**) putting the assembly samples in a hot-pressing machine for the bonding process, and (**f**) obtaining as-fabricated samples after the bonding process.

**Figure 3:**
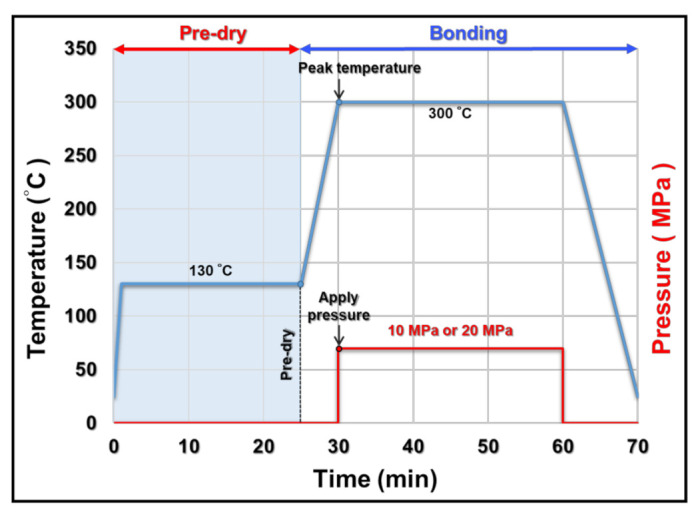
Temperature and pressure profiles for the sintering process.

Figure 2 shows a flowchart for the sample preparation process of the sintered Ag and Ag–In alloy joints. Cu (99.9% purity) substrates were chosen as top and bottom dies. The dimensions of top Cu substrates were 3 mm × 3 mm × 3 mm, and the dimensions of bottom Cu substrates were 10 mm × 10 mm × 3 mm (length × width × thickness). To remove the Cu oxide and create flat surfaces, the top and bottom Cu dies were metallographically polished using SiC abrasive sandpapers (#4000, Struers). Then, a 300 nm thick layer of Ag was sputtered onto the top and bottom Cu substrates as the surface finish layer, as shown in Figure 2a. During the stencil-printing and pre-drying processes shown in Figure 2b,c, respectively, the Ag and Ag–In alloy pastes produced by the ball-milling process were printed onto the bottom Ag-sputtered Cu dies using a 100 μm thick stencil. The pastes were then pre-cured on a hot plate at 130 °C for 25 min to volatilize the organic solvent. Finally, Ag-sputtered top dies were put on the pre-cured pastes, and the sintered Ag and Ag–In alloy joints were fabricated using a hot-pressing bonding machine. The bonding condition for the thermal-compressive bonding process is presented in Figure 3. The atmosphere in the chamber of the hot-pressing machine (Yongfa Technology Company, Taipei, Taiwan) was replaced with inert nitrogen during heating to prevent oxidation. The bonding temperature was set at 300 °C for 30 min with a compressive stress of 10 MPa or 20 MPa to promote bonding. After the bonding process, the bonded samples underwent the furnace cooling process. Finally, the as-bonded samples of sintered Ag and Ag–In alloy joints could be achieved after cooling.

### 2.3. Reliability Tests

**Figure 4:**
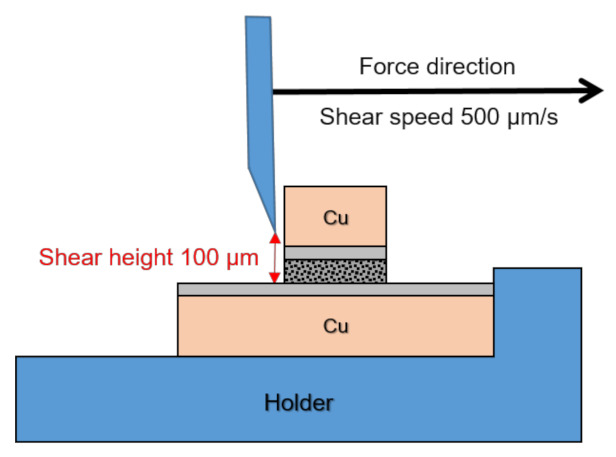
Schematic of the die shear test.

HTS and die shear tests were performed to examine the reliability and mechanical properties of the sintered Ag and Ag–In alloy joints. The HTS test temperature was 300 °C in a furnace with an atmospheric environment to test the short- and long-term high-temperature reliability of both sintered joints. After the HTS test, die shear tests were performed using a shear tester (Condor Sigma Lite, XYZTEC, Panningen, The Netherlands) to measure the shear strengths and conduct the assessment for the mechanical reliability of both sintered joints. Figure 4 depicts the schematic diagram of the die shear test. The distance between the shear tool and the bottom die was 100 μm, and the speed for the die shear test was 500 μm/s.

### 2.4. Phase Identification and Compositional Analysis

The morphology and microstructure of the ball-milled Ag and Ag–In alloy powders were characterized using a scanning electron microscope (SEM, Hitachi SU-5000, Hitachi, Tokyo, Japan) equipped with an energy-dispersive X-ray spectrometer (EDS, Bruker, Berlin, Germany). The phases of the Ag–In alloy powders were identified using X-ray diffraction (XRD, Rigaku TTRAX3, Tokyo, Japan) with Cu Kα radiation (λ = 0.15418 nm). A transmission electron microscope (TEM, FEI Tecnai G2 F20, FEI, Roanoke, VA, USA) was used to observe the crystal structure of the ball-milled powders. In addition, the size distributions of the powders were examined using a laser-diffraction particle size analyzer (Coulter LS230, Miami, FL, USA), with an examination range from 0.04 μm to 2000 μm. After the hot-pressing bonding process and the HTS test, bonded samples were mounted with epoxy resin and polished using abrasive sandpapers. For SEM observation, an artifact-free cross-section was created by using an ion-milling system equipped with an Ar^+^ ion gun (Hitachi IM-4000, Hitachi, Tokyo, Japan). Moreover, an electron probe microanalyzer (EPMA, JEOL JXA-8530FPlus, JEOL, Tokyo, Japan) was utilized for elemental mapping and compositional analysis.

## 3. Results

### 3.1. Characterization of the Ag and Ag–In Alloy Powders after Ball-Milling

**Figure 5:**
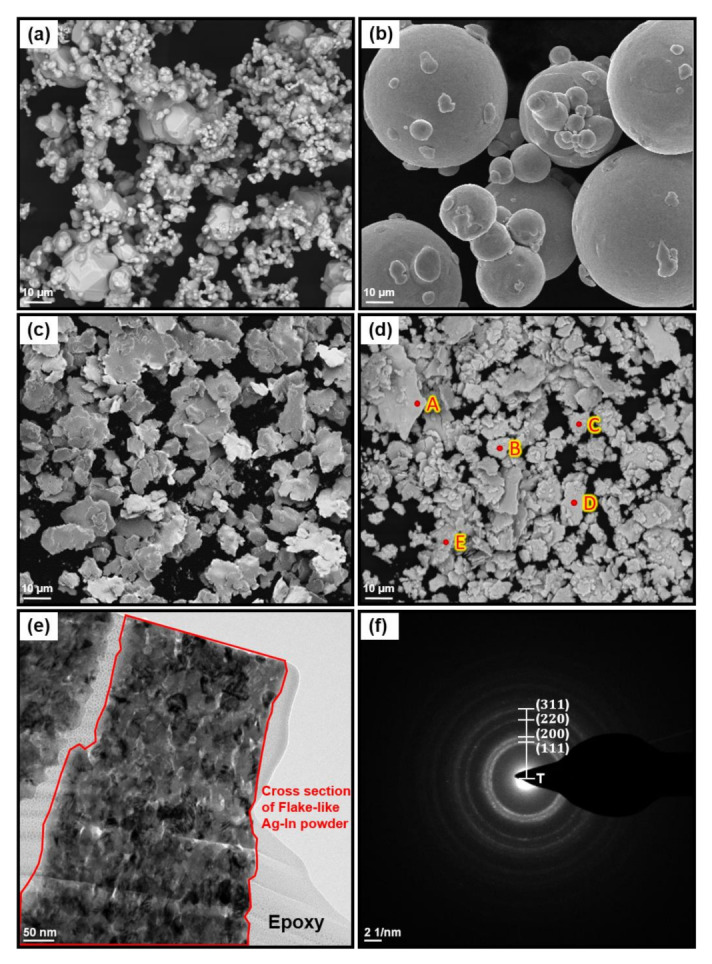
Raw and ball-milled powder characterizations. SEM images of (**a**) the raw Ag powder, (**b**) the raw In powder, (**c**) the ball-milled Ag powder, and (**d**) the ball-milled Ag–In alloy powder. (**e**) TEM images and (**f**) the corresponding TEM diffraction pattern for the ball-milled Ag–In alloy powder.

Figure 5 shows the characterization of the raw Ag, raw In, ball-milled Ag, and ball-milled Ag–In alloy powders. The morphology of both the ball-milled Ag and the Ag–In alloy powders consisted of flakes after milling, and both the ball-milled powders were refined compared to raw powders, as shown in Figure 5a–d. The ball-milled Ag–In alloy powders exhibited nanocrystalline structure from the grain contrast of the bright-field TEM image in Figure 5e. The generation of nanocrystalline domains was attributed to the constant high-energy impact on the Ag and In powders during the ball-milling process, causing the accumulation of numerous grain boundaries within the ball-milled powders. The crystal structure of Ag–In alloy powders was determined to be face-centered cubic (FCC) from the TEM diffraction pattern in Figure 5f. The diffraction ring of the Ag–In alloy powder was similar to that of pure Ag; only the lattice constants were different. On the basis of the TEM ring pattern, the lattice constant of the Ag–In alloy powder was calculated to be 0.414 nm, which was slightly higher than that of the Ag powders (0.409 nm) [29]. The difference in the lattice constant was due to lattice distortion in the Ag–In alloy powder caused by the solid solution of In in the Ag matrix.

**Figure 6:**
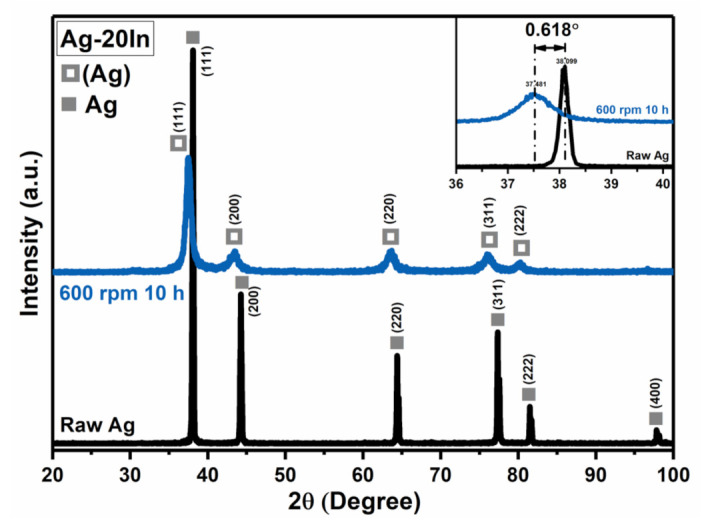
XRD patterns of the Ag–In alloy powders.

The XRD patterns for the raw Ag and Ag–In alloy powders before and after the ball-milling process are shown in Figure 6. This measurement confirms that the crystal structure of the Ag–In alloy powder remained FCC after the ball-milling process. There was no change in the crystalline structure after ball-milling; only the XRD peak position shifted, which suggested a change in the lattice constant. The lattice constant of the Ag–In alloy powder was calculated to be 0.414 nm from the XRD pattern, which was consistent with the TEM analysis. Moreover, no peaks of Ag–In IMCs could be identified from the XRD pattern of the Ag–In alloy powder. This illustrates that a single phase of the Ag–In alloy powder with a homogeneous Ag-based Ag–In solid solution could be obtained via mechanical alloying. In other words, the solid solution and homogenization of the Ag–In alloy powder were achieved through sufficient diffusion between the pure Ag and In powders under such milling conditions. The SEM–EDS compositional analysis of the Ag–In alloy powder in Figure 5d is shown in Table 1. The phase of the Ag–In alloy powder was identified to be Ag-based Ag–In solid solution, labeled (Ag)–In. The elemental analytical result was consistent with the XRD result. TEM–EDS also indicated that the mean composition of the Ag–In alloy powder was 82.3 at.% Ag and 17.7 at.% In, which corresponds with the elemental compositions determined using SEM–EDS.

**Figure 7:**
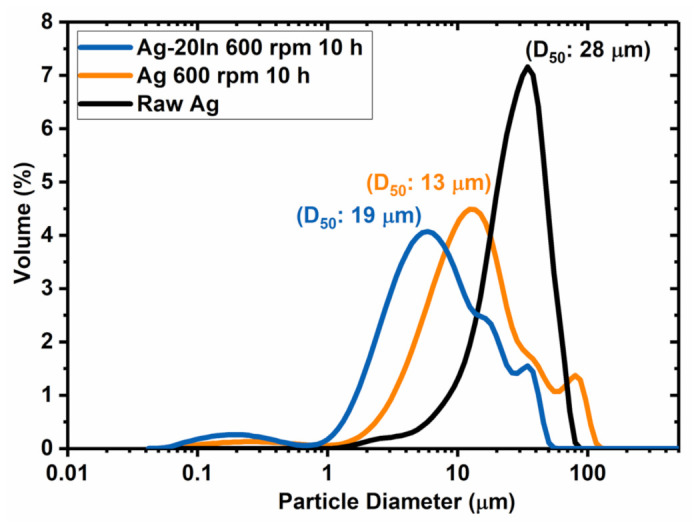
Particle size distributions for the Ag and Ag–In alloy powders after the ball-milling process.

The particle size distributions (PSDs) for the Ag and Ag–In alloy powders after the ball-milling process are shown in Figure 7. In the PSD analysis, the value D_50_ indicates that 50 vol.% of the powder had a diameter less than this value. Herein, we regarded D_50_ as the average particle size and compared the D_50_ of powders before and after ball-milling. The D_50_ of the raw Ag powder was 28 μm, while the D_50_ of the ball-milled Ag and Ag–In alloy powders was 19 μm and 13 μm, respectively. Hence, both the ball-milled Ag and the Ag–In alloy powders were refined through the fracturing effect during the milling. The agglomeration and coarsening of ductile Ag and In powders caused by cold welding could be effectively inhibited by the addition of stearic acid, which was consistent with previous studies [30]. The Ag and Ag–In alloy powders were sieved with a 25 μm mesh after ball-milling. The resulting powders were mixed with the organic solvent to produce the Ag and Ag–In alloy pastes used for sintering and bonding applications.

### 3.2. Microstructure of the Sintered Ag Joints during HTS at 300 °C

**Figure 8:**
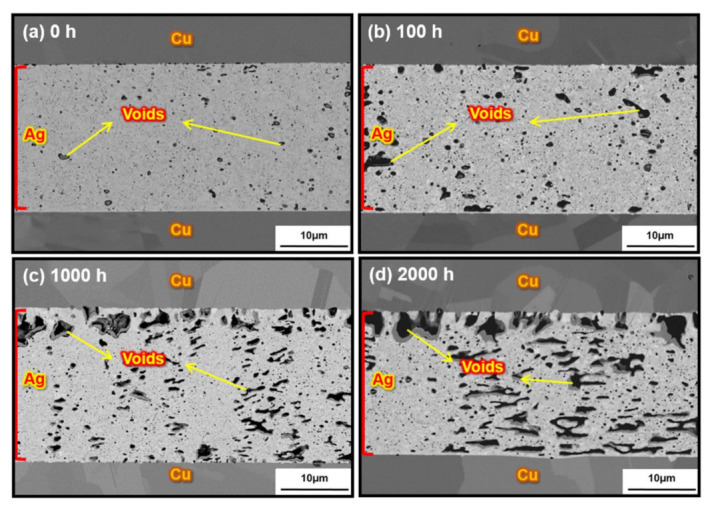
SEM images of the cross-section microstructure of the sintered Ag joints bonded at 10 MPa after HTS at 300 °C for (**a**) 0 h, (**b**) 100 h, (**c**) 1000 h, and (**d**) 2000 h.

Figure 8 shows SEM images of the microstructure cross-sections of the sintered Ag joints bonded at 10 MPa after HTS at 300 °C from 0 h to 2000 h. The porosity of the sintered joint was calculated using an image threshold in ImageJ software. The porosity was averaged across five representative positions at the sintered joint of one sample at the same magnification in SEM. Figure 8a shows the cross-sectional microstructure of the as-bonded Ag joint. After sintering the flake-like Ag particles with a compressive pressure of 10 MPa, a low porosity of 4.1% could be achieved in the as-bonded Ag joint, indicating a dense structure. Moreover, excellent wettability was observed at the interface between the 300 nm thick sputtered Ag layer and the sintered flake-like Ag particles. However, the porosity of the sintered Ag joint significantly increased to 9.3%, 11.3%, and 15.7% after the HTS for 100 h, 1000 h, and 2000 h, respectively, as shown in Figure 8b–d. The sintered pores in the Ag joint tended to coalesce via vacancy and grain boundary diffusion during the HTS to reduce the surface energy and achieve a more thermodynamically stable state. Nevertheless, the coarsening of the sintered pores after the HTS severely deteriorated the wettability of the Ag-sputtered Cu substrates, as shown in Figure 8d. Micro-voids were formed at the Ag/Cu wetting interface because of the accumulation and coarsening of the sintered pores in the high-temperature environment. The coarsening of the sintered pores and the poor wettability in the sintered Ag joint after the HTS led to severe stress concentration problems, which caused deterioration of the mechanical properties.

Cu oxidation above 200 °C should have a considerable effect on the pure sintered Ag joint. Although it is not deeply understood yet, many researchers have discussed that the sintered Ag joint would suffer from Cu oxidation at high temperatures, which detrimentally affects the mechanical properties of sintered Ag joints. However, the Cu oxidation phenomenon was not observed in the sintered Ag joint during the HTS at 300 °C in this study. The main reason is the porosity of the as-bonded sintered Ag joint. There is a positive correlation between the porosity of the as-bonded sintered joint and the occurrence of such high-temperature oxidation, which is described by percolation theory. Specifically, the high porosity of the as-bonded sintered joint facilitates the oxidation process during HTS. Assuming that the pores are compact and uniformly distributed, three different theoretical porosity ranges can be calculated using percolation theory to describe whether external media (e.g., O_2_) can invade the porous structure [31,32,33]. To elaborate, a theoretical porosity of less than 3% results in fully isolated and uniformly distributed pores in the sintered joint that are not exposed to external surfaces, isolating the sintered joint from environmental O_2_. Meanwhile, isolated and interconnected pores coexist at the sintered joint in the theoretical porosity range from 3% to 20%, while fully interconnected pores exist to form a 3D porous network at the sintered joint for a theoretical porosity above 20%. Hence, when the porosity of the sintered joint is beyond or close to this theoretical limit of 20%, the 3D porous network provides abundant pathways for O_2_ to invade and penetrate the sintered joint.

In previous studies, a low-pressure or pressureless Ag sintering process was attempted to reduce the production cost. However, the high porosity occurring from low-pressure bonding resulted in interconnected pores in the as-bonded sintered Ag joint; therefore, O_2_ could easily penetrate the edges of the sintered Ag joint during the HTS. As a result, Cu diffusing from the substrate reacted with O_2_ to form a cuprous oxide (Cu_2_O) layer near the interface between the sintered Ag joint and the Cu substrate owing to the high affinity of Cu and O_2_. By contrast, a relatively high bonding pressure was utilized for the flake-like Ag sintering in this study. As a result, a low porosity of 4.1% was achieved in the as-bonded sintered Ag joint due to densification during the sintering process, and most of the pores in the sintered joint were unconnected and isolated. Consequently, O_2_ was effectively prevented from invading the sintered joint, reacting with Cu, and forming the Cu_2_O layer. Although the porosity of the sintered Ag joint significantly increased during the HTS and oxidation should occur, the tendency of the pores to coalesce instead of maintaining a uniform distribution caused most of the pores to remain isolated despite the relatively high porosity. However, although the oxidation issue during the HTS could be effectively addressed by increasing the applied pressure, the sintered pore coarsening phenomenon in the sintered Ag joint would still cause degradation of the mechanical properties.

### 3.3. Microstructure and Phase Identification of the Sintered Ag–In Alloy Joints during HTS at 300 °C

**Figure 9:**
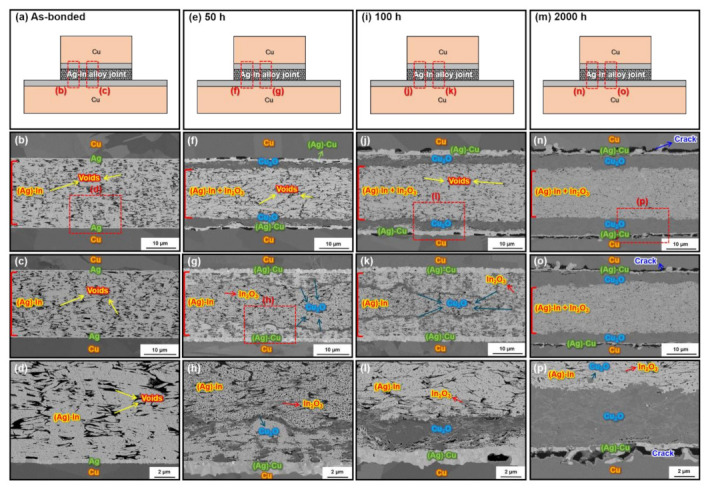
SEM images of the cross-section microstructure of the sintered Ag–In alloy joints bonded at 10 MPa after HTS at 300 °C. (**a**,**e**,**i**,**m**) Schematics of the cross-section of the Ag–In alloy joints as-bonded and after HTS for 50 h, 100 h, and 2000 h, respectively. The dashed boxes in (**a**,**e**,**i**), and (**m**) indicate the regions near the edge shown in (**b**,**f**,**j**), and (**n**), as well as the regions away from the edge shown in (**c**,**g**,**k**), and (**o**), respectively. Zoomed-in images of the regions indicated in (**b**,**g**,**j**) and (**n**) are shown in (**d**,**h**,**l**), and (**p**), respectively.

Figure 9 depicts SEM images of the cross-sectional microstructure of the sintered Ag–In alloy joint bonded at a bonding pressure of 10 MPa during the 2000 h HTS test at 300 °C. The microstructures of the sintered Ag–In alloy joint bonded at 10 MPa were quite different in the side and the central regions of the joint at the early stage of the HTS. Figure 9a,e,i,m indicate the observation positions for the joint after different HTS periods: as-bonded and after 50, 100, and 2000 h, respectively. SEM images of the side (Figure 9b,f,j,n) and central regions (Figure 9c,g,k,o) are shown for the joints. In the same bonding condition, the average porosity of the as-bonded Ag–In alloy joint in Figure 9b,c was 18.9%, which was much higher than that of the as-bonded Ag joint in Figure 8a. This implied that the sinterability of Ag–In alloy powders was worse than that of pure Ag powders. Additionally, micro-voids could be observed at a few regions of the wetting interfaces, as shown in Figure 9d, indicating that the wettability of the as-bonded Ag–In alloy joint was not as good as the as-bonded Ag joint. The composition of the as-bonded Ag–In alloy joint in the sintering zone was also determined to be the homogeneous Ag-based Ag–In solid solution phase from EDS analysis, labeled (Ag)–In in Figure 9b–d.

The porosity of the Ag–In alloy joint did not increase as dramatically as that of the pure Ag joint after the HTS at 300 °C for 50 h and 100 h, as shown in Figure 9f,g and Figure 9j,k, respectively. The porosity of the sintered Ag–In alloy joint was 17.6% and 17.1% after the HTS for 50 h and 100 h, respectively, which was similar to the porosity of the as-bonded Ag–In alloy joint. In other words, the sintered pore coarsening phenomenon was not evident in the Ag–In alloy joint at high temperatures, in contrast to the sintered Ag joint. The most likely reason for the inhibition of the pore coarsening in the Ag–In alloy joint during the HTS was the solute drag effect that restricted the migration of the Ag grain boundaries [34,35,36]. According to the sintering theory, the grain growth is accompanied by the coarsening of sintered pores, and this process is highly dependent on grain boundary diffusion. However, the indium element as the solute alloying in the Ag–In alloy joint would prohibit the movement of grain boundaries. Consequently, the sintered pores could not coalesce via grain boundary diffusion. Hence, the pore coarsening phenomenon during the HTS could be effectively inhibited by adding In. However, severe oxidation issues still occurred in the Ag–In alloy joint bonded at 10 MPa because the porosity of the as-bonded Ag–In alloy joint was close to the percolated porosity limit of 20%.

**Figure 10:**
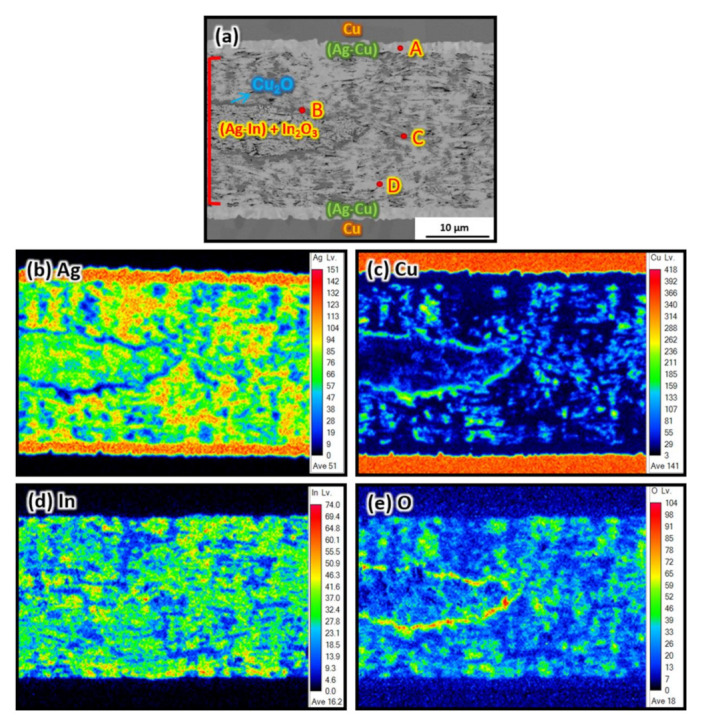
(**a**) Cross-section microstructure of the middle of the Ag–In alloy joint bonded at 10 MPa after HTS at 300 °C for 100 h. (**b**–**e**) Elemental distributions of (**a**).

The EPMA elemental mapping of the Ag–In alloy joint after HTS at 300 °C for 100 h is shown in Figure 10. The elemental content of the area indicated in Figure 10a is listed in Table 2. After the HTS for 50 h and 100 h, a lamellar Cu_2_O layer could be identified between the Ag–In alloy joint and the substrate near the side regions of the joint in Figure 9f,j. In addition, we observed that the Cu_2_O layer converged and ended near the central region of the joint, as shown in Figure 9g,k. This revealed that O_2_ entered the Ag–In alloy joint through the interconnected pores from the edges of the joint. At the beginning of the HTS, the O_2_ concentration was too low to form a lamellar Cu_2_O layer near the central region of the joint. Instead, Cu_2_O clusters with dark-gray contrast were formed around the sintered pores in the central region of the joint, as shown in Figure 9g,h. In_2_O_3_ clusters with light-gray contrast were also found around the sintered pores. However, In_2_O_3_ clusters were identified around the pores in all sintered regions of the joint after the HTS, which was not the case for the Cu_2_O clusters. Furthermore, owing to the interdiffusion of Ag and Cu at the wetting interface, the 300 nm thick sputtered Ag was transformed into the Ag-based Ag–Cu solid solution phase, labeled (Ag)–Cu in Figure 9f,h,j,l,n,p. After the long-term HTS for 2000 h, adequate O_2_ could reach the central region of the Ag–In alloy joint and form a continuous lamellar Cu_2_O layer. The formation and growth of the thick Cu_2_O layers generated cracks at the interface between the continuous Cu_2_O layer and the substrate, which negatively affected the mechanical properties.

**Figure 11:**
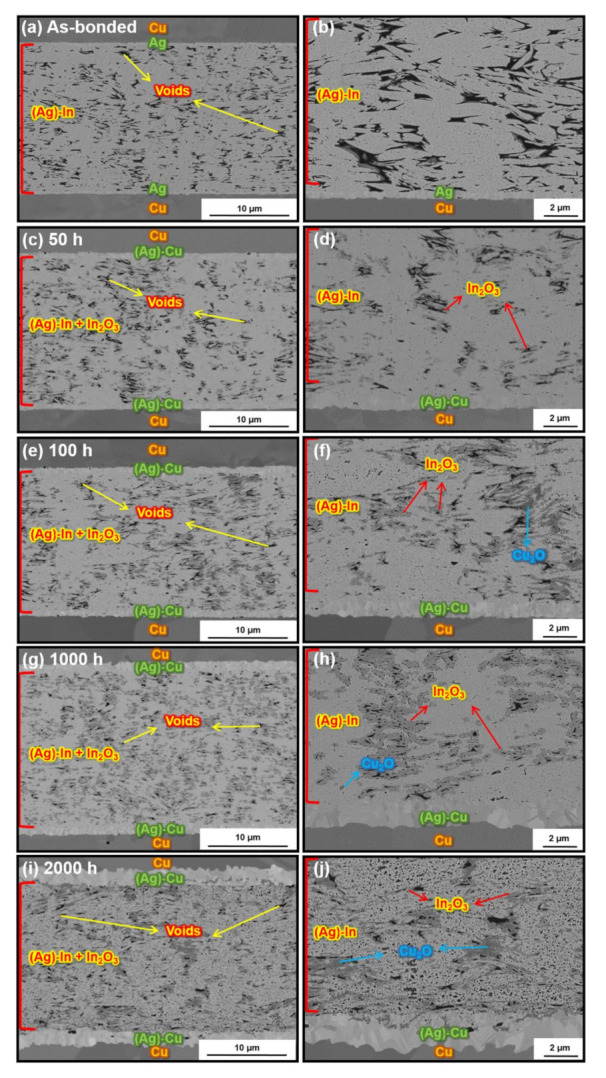
SEM images of the cross-section microstructure of the sintered Ag–In alloy joints bonded at 20 MPa after HTS at 300 °C for the (**a**) as-bonded joints and sintered joints after HTS for (**c**) 50 h, (**e**) 100 h, (**g**) 1000 h, and (**i**) 2000 h. Magnified images of (**a**,**c**,**e**,**g**), and (**i**) are shown in (**b**,**d**,**f**,**h**), and (**j**), respectively.

Figure 11 shows SEM images of the cross-sectional microstructure of the sintered Ag–In alloy joint bonded at a bonding pressure of 20 MPa during the 2000 h HTS test at 300 °C. As shown in Figure 11a, the porosity of this joint was dramatically lower at 10.6%. Compared to the as-bonded Ag–In alloy joint bonded at 10 MPa, not only did the porosity decrease but the wettability also increased at the higher bonding pressure. Although interconnected pores still existed at this porosity according to the predictions of the percolation theory, the relatively low porosity could still reduce the penetration of O_2_. After 50 h to 2000 h of HTS, as shown in Figure 11b–e, no continuous Cu_2_O layer was found in the sintered joint, which indicated that the amount of O_2_ entering the joint bonded at 20 MPa was significantly reduced compared to the joint bonded at 10 MPa. Nevertheless, clusters of Cu_2_O and In_2_O_3_ could still be observed around the sintered pores after the HTS; interconnected pores were still present in the as-bonded Ag–In alloy joint, which provided pathways for O_2_ penetration. Although the Cu_2_O and In_2_O_3_ clusters were formed in the Ag–In alloy joint, the oxide clusters around the sintered pores had a negligible effect on the mechanical properties of the joints compared to the lamellar oxides and cracks. More importantly, the pores in the sintered Ag–In alloy joint bonded at 20 MPa did not coarsen, which was consistent with the results at 10 MPa.

### 3.4. Mechanical Properties of the Sintered Ag and Ag–In Alloy Joints during HTS at 300 °C

**Figure 12:**
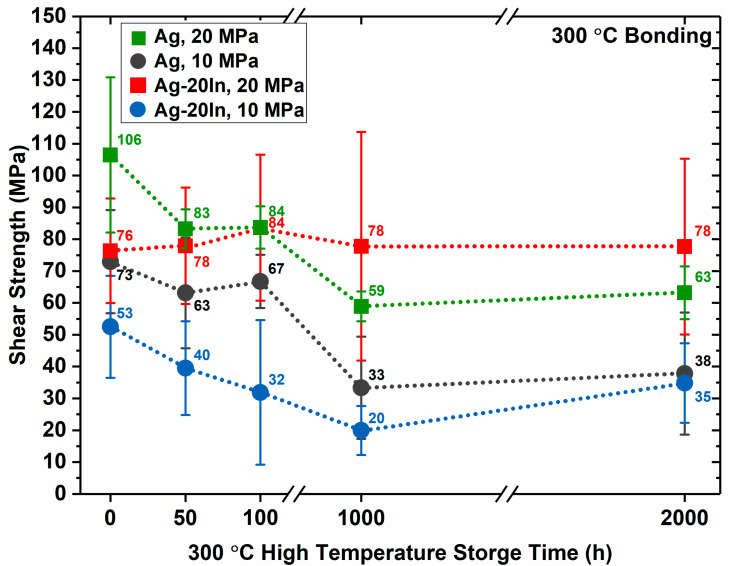
Variation of the shear strength for sintered Ag and Ag–In alloy joints with the thermal aging time at 300 °C.

The shear strength variations of the sintered Ag and Ag–In alloy joints with the HTS treatment time are illustrated in Figure 12. Each data point was averaged over four sintered joints. The gray dashed line in Figure 12 depicts the change in shear strength of the sintered Ag joint at a bonding pressure of 10 MPa during the HTS. The mean shear strength of the as-bonded Ag joint bonded at 10 MPa was 73 MPa, which was higher than that of the as-bonded Ag–In alloy joint with the same bonding condition due to the lower porosity and the better wettability. However, the shear strength of the Ag joint bonded at 10 MPa dramatically decreased after the HTS. The deterioration of mechanical properties was attributed to the sintered pore coarsening and the correspondingly poor wettability, which gave rise to stress concentration that significantly deteriorated the mechanical reliability of the sintered Ag joints. The green dashed line in Figure 12 depicts the change in shear strength of the sintered Ag joints at the bonding pressure of 20 MPa during the HTS. The shear strength of the as-bonded Ag joint bonded at 20 MPa was 106 MPa. This value was better than that of the as-bonded Ag joint bonded at 10 MPa, demonstrating that the mechanical properties can be improved by increasing the bonding pressure. The reinforcement of the sintered Ag joint was attributed to the lower porosity and better wettability. Nevertheless, although the as-bonded sintered Ag joint was strengthened by the higher pressure, the mechanical strength was still compromised during the HTS due to the coarsening of the sintered pores and the wettability issues.

The blue dashed line in Figure 12 illustrates the change in shear strength of the sintered Ag–In alloy joints bonded at 10 MPa during the HTS. The shear strength of the as-bonded Ag–In alloy joints bonded at 10 MPa was 53 MPa, which was slightly lower than that of the as-bonded Ag joints with the same bonding conditions owing to the higher porosity. The mechanical properties of the Ag–In alloy joints bonded at 10 MPa were found to significantly deteriorate during the 2000 h HTS. The reason for the decrease in shear strength of the Ag–In alloy joints was different from that of the sintered Ag joints. The deterioration of the Ag–In alloy joint shear strength was not due to the coarsening of the sintered pores but rather from the formation of continuous Cu_2_O layers and cracks. The red dashed line in Figure 12 illustrates the change in shear strength of the sintered Ag–In alloy joints bonded at 20 MPa during the HTS. The shear strength of the as-bonded Ag–In alloy joints bonded at 20 MPa was better than that of the as-bonded Ag–In alloy joints bonded at 10 MPa owing to the lower porosity at the higher bonding pressure. The mechanical properties of the Ag–In alloy joints bonded at 20 MPa were found to exhibit excellent shear strengths and stable mechanical properties during the 2000 h HTS. The low porosity of the Ag–In alloy joints achieved at a bonding pressure of 20 MPa was sufficient to prevent a large amount of external O_2_ from entering the interior of the sintered joint and forming fragile Cu_2_O layers and cracks. Furthermore, the addition of In was proven to help prevent sintered pore coarsening. Consequently, the Ag–In alloy joint with the optimal bonding condition was the most mechanically reliable at high temperatures because it simultaneously overcame the oxidation and pore coarsening issues.

**Figure 13:**
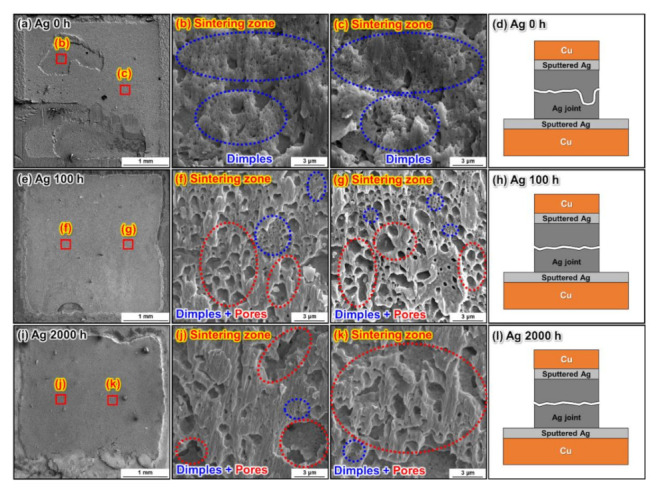
Surface fracture analysis of the sintered Ag joints bonded at 10 MPa. Fracture planes of the sintered Ag joints (**a**) as-bonded and after (**e**) 100 h and (**i**) 2000 h of HTS at 300 °C. (**b**,**c**), (**f**,**g**), and (**j**,**k**) are magnified images of the fracture plane areas marked in (**a**,**e**), and (**i**), respectively. Schematic of the crack propagation in the sintered Ag joints (**d**) as-bonded and after (**h**) 100 h and (**l**) 2000 h of HTS at 300 °C.

The surface fracture analyses of the sintered Ag joints are shown in Figure 13. Large quantities of micro-dimples were formed on the fracture surfaces of the as-bonded Ag joints, indicating a ductile deformation of the joint, as shown in Figure 13b,c. Figure 13d depicts the schematic drawing of the fracture failure pattern of the as-bonded Ag joint; the failure was along the sintering zone in the sintered Ag joint after the die shear test. Although a few dimples could still be observed on the fracture surfaces after the HTS for 100 h and 2000 h, as shown in Figure 13f,g,j,k, the density of dimples on the fracture surfaces gradually decreased as the HTS time increased. This was due to sintered pore coarsening, which led to stress concentration in the sintered Ag joint. Hence, cracks originated from these large pores when subjected to external forces, resulting in the deterioration of the shear strength, a tendency to cleave, and a decrease in the density of dimples on the fracture surfaces. Figure 13h,l show schematics of the fracture failure pattern of the Ag joint after the HTS for 100 h and 2000 h, respectively. Although the sintered pores dramatically coarsened after the HTS, cracks still propagated along the sintering zone in the Ag joint.

**Figure 14:**
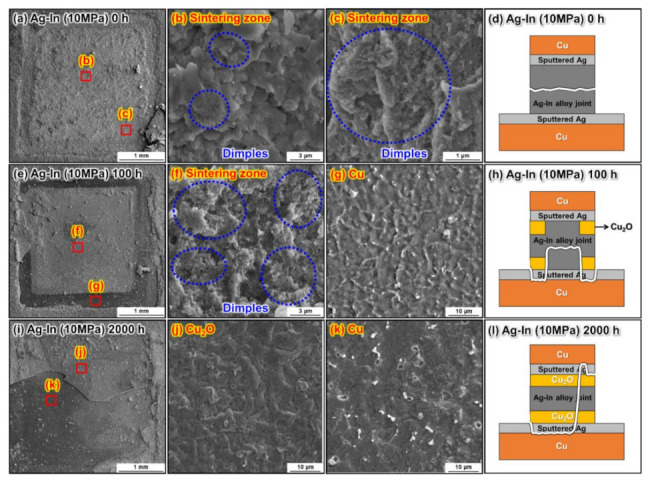
Surface fracture analysis of the sintered Ag–In alloy joints bonded at 10 MPa. Fracture planes of the Ag–In alloy joints (**a**) as-bonded and after (**e**) 100 h and (**i**) 2000 h of HTS at 300 °C. (**b**,**c**), (**f**,**g**), and (**j**,**k**) are magnified images of the fracture plane areas marked in (**a**,**e**), and (**i**), respectively. Schematic of the crack propagation in the sintered Ag–In alloy joints bonded at 10 MPa (**d**) as-bonded and after (**h**) 100 h and (**l**) 2000 h of HTS at 300 °C.

**Figure 15:**
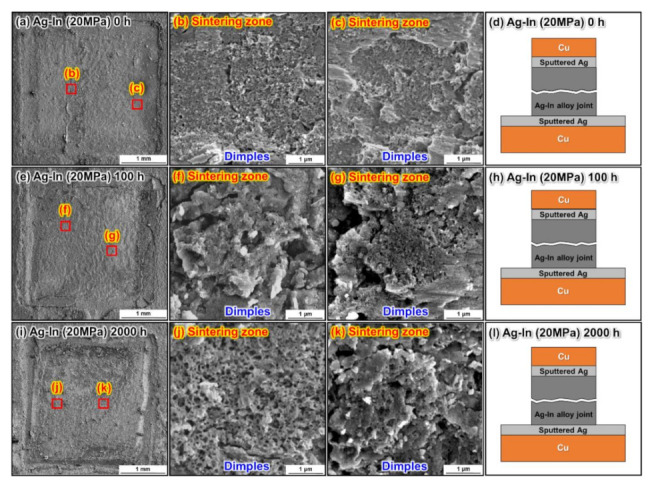
Surface fracture analysis of the sintered Ag–In alloy joints bonded at 20 MPa. Fracture planes of the Ag–In alloy joints (**a**) as-bonded and after (**e**) 100 h and (**i**) 2000 h of HTS at 300 °C. (**b**,**c**), (**f**,**g**), and (**j**,**k**) are magnified images of the fracture plane areas marked in (**a**,**e**), and (**i**), respectively. Schematic of the crack propagation in the sintered Ag–In alloy joints bonded at 20 MPa (**d**) as-bonded and after (**h**) 100 h and (**l**) 2000 h of HTS at 300 °C.

The fracture surface analysis of the sintered Ag–In alloy joints bonded at 10 MPa and 20 MPa is shown in Figure 14 and Figure 15, respectively. Ductile deformation characteristics could be identified on the fracture surfaces of the as-bonded Ag–In alloy joints, as shown in Figure 14a–c and Figure 15a–c. The composition was determined to be (Ag)–In on the fracture surfaces of both as-bonded Ag–In alloy joints. However, Cu surfaces were observed on the fracture surfaces of the Ag–In alloy joint bonded at 10 MPa after the HTS for 100 h, as shown in Figure 14e–g. At the early stages of the HTS, the Cu_2_O layers mainly formed near the edges of the sintered joints. Hence, the Cu surfaces were near the edges of the fracture plane, which was consistent with the cross-sectional SEM analysis. This indicated that cracks tended to propagate along the fragile Cu_2_O layers near the edges, rather than along the sintered joints, as illustrated in Figure 14h. After 2000 h of HTS, continuous Cu_2_O layers were formed throughout the sintered joint, and the cracks only propagated along the Cu_2_O layers, as shown in Figure 14i–l. By contrast, a Cu layer could not be located on the fracture surfaces of the Ag–In alloy joint bonded at 20 MPa after the HTS for 100 h and 2000 h, as shown in Figure 15e–l. The compositions of the fracture surfaces were mainly the (Ag)–In solid solution. This again demonstrated that the oxide clusters had little impact on the mechanical properties, and that the continuous oxide layer was not formed in the Ag–In alloy joint bonded at 20 MPa after the HTS due to the low porosity. Moreover, micro-dimples could be identified on all the fracture planes of the Ag–In alloy joint after the HTS, indicating that the Ag–In alloy joint maintained excellent ductility at high temperatures.

## 4. Discussion

### 4.1. Oxidation Mechanism of the Ag–In Alloy Joint Bonded at 10 MPa

Two types of Cu_2_O and one type of In_2_O_3_ were observed in the Ag–In alloy joint bonded at 10 MPa after the HTS, including a Cu_2_O layer, Cu_2_O clusters, and In_2_O_3_ clusters, as shown in Figure 9. The porosity of the Ag–In alloy joint for this bonding condition was not sufficiently low to completely block external O_2_; therefore, O_2_ could still attack the sintered Ag–In alloy joint through interconnected pores and react with Cu and In. This resulted in the formation of a Cu_2_O layer, which led to severe reliability issues.

**Figure 16:**
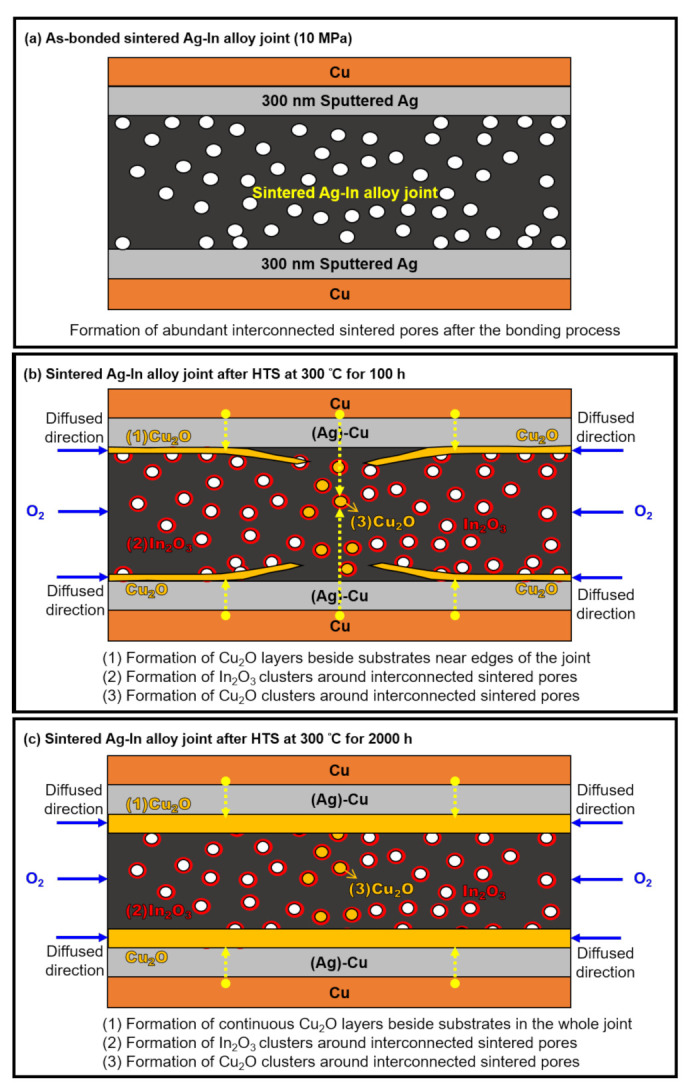
Proposed mechanism for the oxidation process of the sintered Ag–In alloy joints at a bonding pressure of 10 MPa. Schematic drawings for the cross-section of the sintered Ag-In alloy joints after the HTS at 300 °C for (**a**) 0 h, (**b**) 100 h, and (**c**) 2000 h.

Herein, a mechanism for the oxidation process in the sintered Ag–In joint was established, as illustrated in Figure 16. The diffusion of four elements (Ag, Cu, In, and O) in and around the sintered Ag–In alloy joint needs to be considered during the HTS. Initially, O_2_ from the environment entered the Ag–In alloy joint via the interconnected pores at the edges of the joints; the high porosity favors the penetration of O_2_. Consequently, the concentration of O_2_ near the edges would be higher than that near the middle of the joint at the beginning of the HTS. Therefore, continuous Cu_2_O layers were observed between the Ag–In alloy joint and the Ag-sputtered Cu substrate near both edges of the sintered joints, as shown in Figure 16b. Cu entering the Ag–In alloy joint through Cu–Ag interdiffusion from the substrate contacts O_2_ and immediately reacts to form the Cu_2_O layer near the wetting interfaces at both edges of the joint. From the EPMA analysis, it was found that the 300 nm thick sputtered Ag layer was transformed into a Ag-rich Ag–Cu solid solution phase, labeled (Ag)–Cu, which was the evidence for the Ag–Cu interdiffusion during the HTS. In addition, near the middle region of the Ag–In alloy joint at the early stages of the HTS, the convergence of the Cu_2_O layer can be seen, as shown in Figure 16b. This indicates that the external O_2_ was not sufficient to form a Cu_2_O layer in the middle region of the joint at this stage. Therefore, Cu from the substrate needed to diffuse a relatively long distance before it formed the Cu_2_O layer near the middle region of the join, as illustrated in Figure 16b. Cu_2_O also existed in the form of clusters located around the sintered pores in the middle region of the joint. The Cu_2_O clusters were mainly formed there in the early stages of the HTS because O_2_ that had reached the middle region via the interconnected pores was not sufficient to form a continuous Cu_2_O layer. However, Cu_2_O clusters could not be found near the edges of the joint; the Cu_2_O layer caused the Cu diffusing from the substrates to continue to react with O_2_, forming a thick layer. In addition to the two types of Cu_2_O contained in the joint, In_2_O_3_ clusters were found in all sintered regions of the joint after the HTS. In_2_O_3_ clusters precipitated around the sintered pores in the Ag–In alloy joint because of the O_2_, which entered the joint through the interconnected pores, similar to the case for the Cu_2_O clusters. Additionally, mixed In_2_O_3_ and Cu_2_O clusters precipitated in the middle region of the joint because O_2_ reacted with both In from the joint and Cu diffusing from the substrates. After the long-term HTS in Figure 16c, continuous Cu_2_O layers and cracks were formed at both interfaces between the Ag–In alloy joint and the substrate. At this stage of the HTS, a considerable amount of O_2_ diffused into the middle of the joint over a long period, causing the Cu_2_O layers near the edges to merge and form continuous Cu_2_O layers. Having developed a complete understanding of the oxidation mechanism in the sintered joint, it is now important to find solutions for overcoming oxidation issues.

### 4.2. Strategies to Overcome Oxidation and Enhance Mechanical Properties of Sintered Joints at High Temperatures

Sintered nano- and micro-Ag materials are promising for die attachment of power chips. In recent years, many studies have focused on pursuing low-pressure or pressureless bonding processes for Ag sintering to achieve cost reductions. However, the porosity of the sintered joint would unavoidably increase when a low-pressure or pressureless bonding was attempted. Consequently, this would significantly increase the risk of high-temperature oxidation, which affects the mechanical properties of the sintered joint. Therefore, developing a strategy for overcoming oxidation problems is a critical and urgent issue for sintered joints. Meanwhile, it is also crucial to inhibit the sintered pore coarsening phenomenon at high temperatures because the pore coarsening also decreases mechanical strength. Alloying the Ag joint has become the primary strategy to combat this issue in pure sintered Ag joints.

In this study, we found a positive correlation, described by percolation theory, between the porosity of the as-bonded sintered joint and the occurrence of such high-temperature oxidation. In other words, the sintering porosity of the as-bonded sintered joint is an important reliability evaluation metric for high-temperature oxidation. According to the predictions of the percolation theory, high-temperature oxidation can be completely prevented when the porosity is below approximately 3%. Hence, reducing the porosity of the as-bonded sintered joint as much as possible helps prevent high-temperature oxidation. Previous studies have proven that the TLP bonding reaction can help prevent oxidation because the introduction of low-melting-point liquid metals during bonding can fill the gaps between the nano- and micro-Ag particles and form isolated pores. This not only resolves the oxidation problem but also addresses the sintered pore coarsening phenomenon. However, the TLP bonding method is not favored in industrial applications. Hence, in this study, the sintering behavior of the Ag–In alloy paste developed by ball-milling was investigated. The results demonstrated that increasing the bonding pressure is another effective method to lower the porosity. The low-porosity Ag–In alloy joint with the optimized bonding condition exhibited excellent reliability because it simultaneously avoided the oxidation and sintered pore coarsening problems. Consequently, the Ag–In alloy joint could retain its outstanding mechanical properties at high operating temperatures. In addition to reducing the porosity, enhancing the heat dissipation of power chip modules is another way to avoid oxidation. If the heat dissipation is sufficient to keep the working temperature from exceeding 200 °C, then the oxidation problem can also be avoided because the driving force to form the Cu_2_O layer in the sintered joints is insufficient. Lastly, another potential strategy is to introduce a surface finish as a diffusion barrier to prevent the diffusion of Cu from the substrate at high temperatures.

## 5. Conclusions

**Figure 17:**
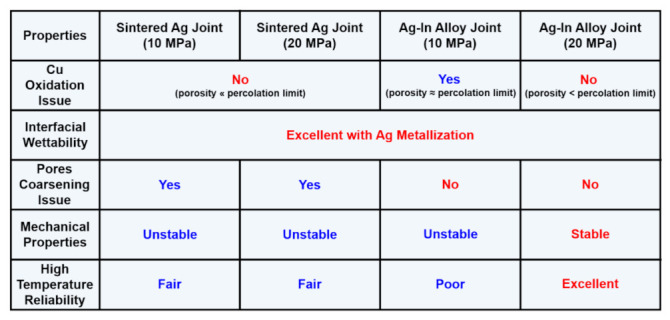
Graphical summary of the benefits of robust Ag–In alloy joints bonded with 20 MPa compared to other joints in 300 °C high-temperature applications.

In this study, pure Ag and In powders were refined to make flake-like Ag and Ag–In alloy powders using a high-energy ball-milling process. Homogeneous Ag–In alloy powders in solid solution could be obtained via mechanical alloying. Moreover, Ag and Ag–In alloy pastes containing particle sizes less than 25 μm were synthesized for power chip attachment after sieving and mixing with an organic solvent. The graphical summary of the sintered Ag joints and Ag-In alloy joints is exhibited in Figure 17. The Ag joint bonded at 10 MPa exhibited excellent mechanical properties. In comparison to low-pressure or pressureless Ag bonding in previous studies, the Ag joint bonded at an optimized pressure significantly reduced the porosity, preventing the joint from being oxidized at high temperatures. However, the well-bonded Ag joint with low porosity still could not avoid the sintered pore coarsening issue in a high-temperature application environment, which significantly deteriorated the mechanical properties. Conversely, the as-bonded Ag–In alloy joint bonded at 10 MPa exhibited outstanding mechanical properties with a shear strength of 53 MPa as well. Sintered pore coarsening was inhibited in the Ag–In alloy joint during the HTS. However, the Ag–In alloy joint bonded at 10 MPa suffered from oxidation problems because it had relatively high porosity and interconnected pores, which provided pathways for O_2_ to enter the joint from the edges and deteriorated the sintered joint. Increasing the bonding pressure to 20 MPa significantly reduced the porosity of the Ag–In alloy joint, blocking external O_2_ at high temperatures. Therefore, the robust Ag–In alloy joint possessed the most stable shear strength and reliable mechanical properties by maintaining high shear strength of above 76 MPa without deterioration in a high-temperature environment because it inhibited the sintered pore coarsening and oxidation issues simultaneously. Lastly, the mechanism for the oxidation process in sintered joints was established in this study and used to formulate strategies to prevent such oxidation. In conclusion, a novel ball-milling method was employed in this study to produce the Ag and Ag–In alloy pastes for die attachment through mechanical alloying, and a promising and reliable Ag–In alloy material was developed for power electronic packaging. It is considered that the materials and technologies developed in this study have great potential for practical application in power-chip attachment and can meet the urgent requirements of future industries for highly reliable power semiconductor packaging materials for EVs or aerospace technology.

## Figures and Tables

**Table 1:** Composition determined using EDS at the positions indicated in Figure 5d.

Position	Ag (at.%)	In (at.%)	Phase
A	81.1 ± 0.26	18.9 ± 0.26	(Ag)–In
B	81.2 ± 0.26	18.8 ± 0.26	(Ag)–In
C	81.9 ± 0.26	18.1 ± 0.26	(Ag)–In
D	81.8 ± 0.26	18.2 ± 0.26	(Ag)–In
E	81.7 ± 0.26	18.3 ± 0.26	(Ag)–In

**Table 2:** Composition determined using EPMA at the positions indicated in Figure 10a.

Position	Ag (at.%)	In (at.%)	Cu (at.%)	O (at.%)	Phase
A	92.6	0	7.4	0	(Ag)–Cu
B	7.8	2.9	58.2	30.1	Cu_2_O
C	0.9	38.2	1.2	59.7	In_2_O_3_
D	86.7	9.4	2.7	1.2	(Ag)–In

## Data Availability

Not applicable.
